# Prophylaxis of Typhoid Fever

**Published:** 1876-04

**Authors:** 


### PROPHYLAXIS OF TYPHOID FEVER.

For a long time infectious diseases have been divided into two principal classes—the contagious and the miasmatic. By *contagium* is meant a poison that arises in the body of one person and is capable of being transmitted from that person to another, either by direct contact, a close proximity to the affected patient, by infected clothing or through the medium of a third person. *Misam* is a poison that is generated and reproduced outside of the human body and infects those brought under its influence. But as the poison is not reproduced in the body of the infected person, he cannot infect a second person.

Among the diseases recognized as infectious, or originating through the infection of the system with a specific poison, are some that do not properly belong to either of the two classes above mentioned, and yet present some of the characteristics peculiar to each. To this third class has been given, by Liebermeister, the name of *Miasmatic-contagious* diseases. The germs of the poison which produce these diseases seem to originate in the body of the person affected, but the disease cannot be conveyed by direct contact from that person to another. Before the poison can produce its specific effect, it must pass through two stages of developement; the first, that of incubation and germination in the body of the diseased person—the second, that of development outside of the body. To this class of diseases belongs typhoid fever, for a long time regarded as a contagious disease—but concerning which later developments show it is not directly transmissible from patient to patient. Evidence of this fact is not lacking, but is found in the experience of the private practitioner and in the history of hospital management. It is unusual for the attendants of a typhoid fever patient to have the disease themselves—it is the exception instead of the rule—yet there is a specific poison which produces the disease. These organisms are contained in the fresh discharges of a typhoid fever patient. As from the fact that the attendants and nurses of typhoid patients rarely are infected, we reason that the disease is not propagated by direct contact from person to person, and that the poison must be looked for in the excretions, so from the same fact and because the fresh excrements may be freely handled by the attendants without producing infection, the deduction is drawn that the organisms, though contained in the fresh discharges, are in a stage of development, when, if introduced into the body of a healthy person they do not reproduce themselves, and can cause no infection.

Before being capable of this, they must pass through a second stage of development outside of the body. This occurs when the discharges remain for some time standing, and the change is hastened when they are brought in contact with quantities of readily decomposing substances, as in water closets, dung heaps, sewers, cess-pools, and soil damp and rich in organic debris. In this stage of development the poison is in a condition to multiply further in the human body, and produce disease. This second stage of development is accelerated by conditions favorable to the development of the poison, such as were mentioned above, but it nevertheless takes place if the excretions are left to themselves, as in soiled linen and bedding.

This poison retains its vitality for a long time and frequently is carried from place to place by streams of water that have been infected.

With a knowledge of the above facts it is easy to understand what prophylactic measures are necessary for the prevention of this disease. As the dejections become the medium of contagion when they have for a time been exposed to favorable influences, it is of the utmost importance that they should be disposed of in a manner to prevent the second stage of development in the poison they contain, and in such places as shall make it impossible for infection to spread. It is quickly seen that privy vaults, cess-pools and sewers are the most improper and dangerous receptacles of this material, for they present the most favorable conditions for the rapid development of the poison, and at the same time, from their extensive use, a large number of persons are exposed to their emanations. It is also frequently seen that wells are located in close proximity to these refuse depositories, and receive the foul matter which soaks from them into the earth. In the same way streams of water may become infected as is shown by the following instance, narrated by Haegler.

"The village of Lausen, since the passage of the allied armies in 1814, had not suffered from an epidemic of typhoid. Isolated cases introduced from Basle had never spread the infection. During the last seven years there had been no cases at all of typhoid. On the 7th of August, 1872, ten inhabitants were attacked with typhoid fever, and in the nine days following, fifty-seven persons were seized. The epidemic lasted till October.—Out of eight hundred inhabitants of the village, one hundred and thirty were taken sick, almost 17 per cent. Nearly one hundred of these cases commenced during the first three weeks of the epidemic. In addition, seven persons temporarily living in the village, were attacked, most of them some time after their departure from it. The disease appeared only in those houses which were supplied with drinking water from running streams. The houses which only used well water escaped. It was demonstrated that the privy and dung-hill of a house at some distance discharged their contents into a little stream. This stream had a subterraneous communication with the sources of the streams that ran into the village. In this house, on July 10th, a man was attacked with typhoid, and in July there were three more cases in the same house."

Another case related by V. Gietl, shows the manner of the infection and the long vitality of the poison. "A villager who had contracted typhoid fever returned to her native village, a place where typhoid had not existed for several years. The excrements of this person were thrown on the dung-hill. Several weeks later five persons were employed to remove the dung-hill. Of these five, four were attacked with typhoid fever, and one with gastric symptoms and swelling of the spleen. The excrements of these five persous were buried deep in the dung-hill. Nine months later, two persons were employed in completely removing this dung-hill. One of them was attacked with typhoid fever and died of it."

Instances similar to the above can be multiplied almost indefinitely, and they teach us what must be done to prevent infection of healthy persons by typhoid patients. First, all discharges should be thoroughly disinfected. The dejections, and matter vomited should be received into vessels containing some reliable disinfectant, as a strong solution of iron, and should be at once buried in dry soil, at a distance from wells, privies, cess-pools, or streams of water, and the vessels, when emptied, should be scalded and rinsed with more of the disinfecting material. Any of the clothing of the patient, that may become soiled, should be at once disinfected, thoroughly boiled and washed, without allowing it to stand, and the same treatment should be applied to the bedding and clothing, when it is changed, as it should be, every day or two, if possible.

Acting upon the above theory, and observing proper precautions, will retard the propagation of the typhoid poison, and prevent disastrous epidemics of the disease. H.

---

Observe the amount of information afforded the physician and the family, in this number of the BISTOURY, for the small sum of 55 cents a year.

